# Comprehensive comparison of three commercial human whole-exome capture platforms

**DOI:** 10.1186/gb-2011-12-9-r95

**Published:** 2011-09-28

**Authors:** Asan, Yu Xu, Hui Jiang, Chris Tyler-Smith, Yali Xue, Tao Jiang, Jiawei Wang, Mingzhi Wu, Xiao Liu, Geng Tian, Jun Wang, Jian Wang, Huangming Yang, Xiuqing Zhang

**Affiliations:** 1 Beijing Genomics Institute at Shenzhen, 11F, Bei Shan Industrial Zone, Yantian District, Shenzhen 518083, China; 2 Beijing Institute of Genomics, Chinese Academy of Sciences, No.7 Beitucheng West Road, Chaoyang District, Beijing 100029, China; 3 Graduate University of Chinese Academy Sciences, 19A Yuquanlu, Beijing 100049, China; 4 The Wellcome Trust Sanger Institute, Wellcome Trust Genome Campus, Hinxton, Cambridge CB10 1SA, UK

## Abstract

**Background:**

Exome sequencing, which allows the global analysis of protein coding sequences in the human genome, has become an effective and affordable approach to detecting causative genetic mutations in diseases. Currently, there are several commercial human exome capture platforms; however, the relative performances of these have not been characterized sufficiently to know which is best for a particular study.

**Results:**

We comprehensively compared three platforms: NimbleGen's Sequence Capture Array and SeqCap EZ, and Agilent's SureSelect. We assessed their performance in a variety of ways, including number of genes covered and capture efficacy. Differences that may impact on the choice of platform were that Agilent SureSelect covered approximately 1,100 more genes, while NimbleGen provided better flanking sequence capture. Although all three platforms achieved similar capture specificity of targeted regions, the NimbleGen platforms showed better uniformity of coverage and greater genotype sensitivity at 30- to 100-fold sequencing depth. All three platforms showed similar power in exome SNP calling, including medically relevant SNPs. Compared with genotyping and whole-genome sequencing data, the three platforms achieved a similar accuracy of genotype assignment and SNP detection. Importantly, all three platforms showed similar levels of reproducibility, GC bias and reference allele bias.

**Conclusions:**

We demonstrate key differences between the three platforms, particularly advantages of solutions over array capture and the importance of a large gene target set.

## Background

Identifying genetic alterations underlying both rare and common diseases, and also other phenotypic variation, is of particular biological and medical relevance. Even after a decade's effort by the genetics research community since the completion of the first human genome sequences [1,2], most genetic mutations underlying human diseases remain undiscovered. For example, the causative mutations for more than half of human rare diseases [3], the genetic architecture of most common diseases [4,5] and the roles of somatic mutations in most cancers [6] have yet to be characterized. Whole genome re-sequencing can potentially identify these uncharacterized mutations, and in the past few years great strides have been made in this regard with massively parallel DNA sequencing technologies that can be applied to the whole genome [7-10]. However, the cost of these technologies remains too high for them to be used as a standard method. Recent integration of targeted exome capture with massively parallel sequencing to selectively re-sequence the best-understood functional parts of the human genome - comprising less than 2% of protein-coding sequences - provides an effective and affordable alternative to identify some of these causative genetic changes.

Several platforms for human exome capture for massively parallel sequencing have been developed and marketed to date [11-14]. In principle, these platforms fall into three classes: DNA-chip-based capture [11,12], DNA-probe-based solution hybridization [14], and RNA-probe-based solution hybridization [13]. These platforms have resulted in great success in pioneering studies hunting for variants causing rare human diseases [11,15-21], and have also been adopted in efforts towards deciphering human common disease and cancer genomes. Yet questions remain about which of these platforms is best for a given application. For example, how many human genes are targeted by each approach and how even is their coverage? How do capture efficacy, technological reproducibility and biases among the different platforms compare? How much input DNA is required and how convenient is each experimentally? How does the cost-effectiveness compare? And what is the power and accuracy of SNP calling, especially for medically important rare SNPs? Up till now, publicly accessible explorations of methodology have been limited to proof-of-concept studies [11,13,14,22], reviews [23,24], or comparisons carried out on only a subset of genes rather than at the whole-genome level [25].

To provide the community with a more solid means to determine the best platform for their experimental needs, we have performed a comprehensive comparison of three commercial human exome capture platforms: NimbleGen's Sequence Capture Array (Human Exome 2.1 M Array, Roche-NimbleGen), NimbleGen's SeqCap EZ (v1.0, Roche-NimbleGen), and Agilent's SureSelect (Human All Exon Kits, Agilent). Each of the three platforms represents one of the classes of exome capture technology currently available. To assess performance with regard to key parameters, including reproducibility, we conducted deep exome capture sequencing for each platform with two technical duplicates (>30× and >60× coverage) using DNA derived from a cell line from a previously sequenced Asian individual [26]. Other key performance parameters characterized here include the genes targeted, the efficacy of exome capture (including specificity, uniformity and sensitivity), technological biases, and the power and accuracy of exome capture data for subsequent SNP calling. Our findings provide comprehensive insights into the performance of these platforms that will be informative for scientists who use them in searching for human disease genes.

## Results

### Human exome capture with the three platforms

We chose platforms that allowed a comparison of the three different methods currently in use for exome capture. The platforms are based on a chip-hybrid method (NimbleGen Sequence Capture Array) or a solution-hybridization method (NimbleGen SeqCap EZ) with a common set of DNA probes, and a solution hybridization method with RNA probes (Agilent SureSelect). The test DNA sample was from a cell line derived from the individual used in the YanHuang whole-genome sequencing analysis [26], allowing comparison with the existing high-coverage genome sequence.

We sought to comprehensively compare the performance of the three exome capture platforms using the best protocols and experimental design for each. We therefore optimized the standard library construction protocols for all three platforms (see Materials and methods): we minimized the input DNA to 10 μg, 3 μg, and 3 μg for Sequence Capture Array, SeqCap EZ and SureSelect, respectively, and set pre-capture PCR to four cycles and post-capture PCR to ten cycles for all three platforms. We included duplicates for each technique to ensure the reliability and assess the reproducibility of data production. We thus constructed a total of six libraries for the three platforms and used the HiSeq2000 to initially produce >30-fold coverage of unique mapped paired-end 90-bp reads (PE90) for each library. We further sequenced one of the two replicates for each platform to >60-fold coverage to obtain a combined coverage of approximately 100-fold for the purpose of discovering the impact of sequence depth on genotype calling for each of the platforms.

### Targeted genes and coverage

One intrinsic feature of exome capture is its capacity for simultaneous interrogation of multiple targets depending directly on the genes targeted by the capture probes. We first compared the targeted genes and their coverage among the three platforms. As the two platforms (array and EZ) developed by NimbleGen shared a common set of targets, we only needed to compare the Agilent and one NimbleGen platform. We annotated protein-coding genes using a merged dataset of 21,326 genes from the CCDS database (release of 27 March 2009), refGen (release of 21 April 2009) and EnsemblGen databases (release 54), and microRNA genes using 719 genes from the human microRNA database (version 13.0). We also included the 200-bp most-flanking regions from both ends of the targeted sequences: typically, 200-bp flanking regions are co-captured with capture libraries constructed from 200- to 250-bp fragments.

The two target sets were 34.1 Mb (NimbleGen) and 37.6 Mb (Agilent) in size, and shared 30 Mb of targets in common, leaving 4.1 Mb specific to NimbleGen and 7.6 Mb specific to Agilent (Table S1 in Additional file 1). Correspondingly, although both target sets contain similar percentages of functional elements (exomic, >71%; intronic, >24%; and others, <5%), Agilent covered approximately 1,000 more protein-coding genes and approximately 100 more microRNA genes (17,199 protein coding genes, 80.6% of the database total; 658 microRNA genes, 91.4%) than NimbleGen (16,188 protein-coding genes, 75.9%; 550 microRNA genes, 76.5%) (Table S2 in Additional file 1). Of those protein-coding genes, 15,883 overlapped between NimbleGen and Agilent, while 305 were unique to NimbleGen and 1,316 were unique to Agilent. Further analyses showed no over-representation of any class of annotated disease genes in the NimbleGen- or Agilent-specific genes (Table S3 in Additional file 1). In addition, both included roughly 1.6 transcripts per gene, a value consistent with the average number of transcripts per gene in the RefSeq database. The results indicated that the majority of known human genes and their splice alternatives were well accounted for in both capture probe designs.

We assessed the coverage of the protein-coding sequences (CDs) by the two platforms, and again, Agilent-targeted regions showed much better coverage (72.0% of targeted genes with >95% CDs, and 78.5% with >90% CDs) than NimbleGen's (46.1% of targeted genes with >95% CDs, and 61.5% with >90% CDs) (Figure S1 in Additional file 2). However, when including the flanking regions, the coverage was much more improved for NimbleGen (74.2% targeted genes with >95% CDs and 76.0% with >90% CDs) than for Agilent (82.0% targeted genes with >95% CDs and 83.0% with >90% CDs) (Figure S1 in Additional file 2). This reduced the gap in CD coverage rate (from >17% to <8%) between the two analysis sets and indicated a more important role of flanking region capture for NimbleGen.

To obtain more detailed information about the target coverage of these two systems, we looked specifically at their ability to interrogate human disease genes using four known data sets (see below). Of the 5,231 unique genes collected from the Online Mendelian Inheritance in Man database (OMIM; release of 10 March 2011), Human Gene Mutation Database (HGMD; Professional 2009.2), and Genome-Wide Association Study (GWAS; release of 3 March 2011) and Cancer Genome Project (CGP; release of 1 December 2010) databases, Agilent targeted 4,871 with 86% of genes having >95% of CDs covered, in comparison with NimbleGen's 4,642 genes with 83% of genes and >95% of CDs covered (Figure S2 in Additional file 2). Thus, for the current pool of disease genes, both could interrogate most known genes, especially those linked to rare diseases, for which 85% of known causative mutations occur in CDs. This makes both capture methods especially attractive for rare disease gene identification and analysis.

### Exome capture specificity

To assess the extent of exome enrichment, we compared the capture specificity of the three platforms, which was defined as the proportion of reads mapping to target regions. For the two replicates of each platform, we obtained a total of 26 to 80 million filtered reads (2.2 to 7.2 Gb; Table 1), roughly corresponding to >30- and >60-fold coverage of the targeted regions. We mapped these reads to the human genome (hg18) using the strategy described in the Materials and methods. Although the overall proportion of filtered reads that could be mapped (78.8 to 86.4%) or uniquely mapped (69.2 to 82.8%) to the human genome differed between the six replicates, the proportions of reads mapped uniquely to targeted regions were more comparable (54.2 to 58.1%) among the three platforms (Table 1). We also found the percentages of uniquely mapping reads were further improved (by up to 12%) for the two NimbleGen platforms by the inclusion of 200-bp flanking regions in the analyses (for the Agilent platform, this was only 2%). Thus, the final percentage of usable reads was 66.6% for the two NimbleGen platforms but was <60% for the Agilent platform. These results indicated that there is a general comparability of capture specificity for targeted regions among the three platforms if the mapping method does not include the flanking region sequences. However, under mapping procedures where researchers do include this information, the NimbleGen platforms perform better.

**Table 1:** Capture specificity of the three human exome capture platforms

	Filtered			Mapped to genome		Uniquely mapped to genome		Uniquely mapped to TR		Uniquely mapped to TFR	
Replicate^a^	Reads (M)	Read length	Bases (Mb)	Percent of reads	Bases (Mb)	Percent of reads	Bases (Mb)	Percent of reads	Bases (Mb)	Percent of reads	Bases (MB)
NA_r1	37	PE90	3,352	85.2	2,793	81.4	2,682	53.5	1,437	63.1	2,064
NA_r2	58	PE90	5,210	79.3	4,115	76.0	3,944	56.4	2,370	67.9	3,481
NS_r1	31	PE90	2,781	86.4	2,402	82.3	2,287	54.2	1,192	67.7	1,860
NS_r2	80	PE90	7,230	85.9	6,163	82.8	5,964	55.0	3,175	67.6	4,787
AS_r1	26	PE90	2,220	84.4	1,868	74.4	1,645	58.1	1,146	60.1	1,332
AS_r2	66	PE90	5,720	78.8	4,496	69.2	3,950	54.6	2,776	56.4	3,225

^a^AS, Agilent solution; NA, NimbleGen array; NS, NimbleGen solution; r1 and r2 are two replicate experiments for each platform. TFR, targeted and flanking regions; TR, targeted regions.

### Uniformity of coverage

**Figure 1:**
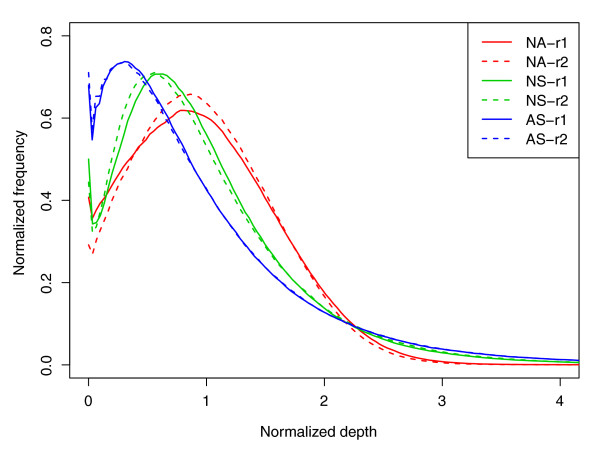
**Normalized per-base sequencing-depth distribution on targets**. For the purpose of comparison among the three platforms, we selected a set of reads with an average coverage of approximately 30-fold from each replicate. The depth and the frequency (the fraction of a certain depth-level bases for certain sequencing depth-coverage in the total sequencing data) were normalized by the average coverage depth of each replicate on targets. NA-r1 and NA-r2, NS-r1 and NS-r2, and AS-r1 and AS-r2 represent each of two replicates for NimbleGen Sequence Capture Arrays, NimbleGen SeqCap EZ and Agilent SureSelect, respectively.

The uniformity of sequence depth over targeted regions determines the genotype sensitivity at any given sequence depth in exome capture. The more uniform the sequencing depth on the targeted region is for a platform, the lower the depth of sequencing that is required to obtain a desired genotype sensitivity. To assess this important quality metric, we selected and analyzed a similar number of reads (approximately 25 million filtered reads, on average approximately 30-fold coverage) from each of the six replicates (Table 2). We found that although all three platforms showed high coverage of their own targeted regions at low sequencing depth (98 to 99% with >1×), the Agilent platform showed more bias towards very low and very high coverage (21% with <10×, 20% with >50×) than the two NimbleGen platforms (<15% with <10×, 7% with >50×). As a result, the two NimbleGen platforms had 10 to 15% more targeted regions (70 to 74%) within 10× to 50× coverage than the Agilent platform (59%). This observation was further supported when we looked at the normalized single base sequencing depth distribution (Figure 1). The curve of the two NimbleGen platforms showed less skew to low and high coverage depths, and more evenness around the mean coverage (approximately 30×), than that of the Agilent platform; that is, the NimbleGen Array showed the best evenness. In addition, the two NimbleGen platforms also showed better uniformity of coverage in flanking regions (Table 2), which is consistent with their better efficiency of capture seen when including the flanking region sequences (Figure S3 in Additional file 2). Thus, the two NimbleGen platforms had a better overall uniformity of sequencing depth than Agilent, which would be expected to impact the relative genotype sensitivity when considering all targets.

**Table 2:** Uniformity of depth by three human exome capture platforms

	Filtered		Mean coverage (×)		Coverage depth (percent of bases in TR)				Coverage depth (percent of bases in FR)			
Replicate^a^	Reads (M)	Bases (Mb)	On TR	On FR	0×	1× to 10×	10× to 50 ×	>50×	0×	1× to 10×	10× to 50×	>50×
NA-r1	25.5	2,280	30.8	9.3	1.3	12.3	69.8	16.6	6.8	56.2	36.8	0.2
NA-r2	27.1	2,421	30.0	8.5	1.0	11.2	73.8	14.1	7.4	53.1	33.1	0.1
NS-r1	26.1	2,338	30.1	10.8	1.7	13.0	69.7	15.6	6.1	53.1	39.5	1.3
NS-r2	25.5	2,289	30.3	9.9	1.5	13.6	68.5	16.3	6.7	56.4	35.8	1.0
AS-r1	25.7	2,222	32.7	3.5	2.1	18.6	59.5	19.8	46.8	42.7	10.1	0.4
AS-r2	25.1	2,175	32.5	3.4	2.2	19.0	59.0	19.8	47.3	42.4	9.9	0.4

For the analyses, a set of data that has 30-fold coverage on targeted regions was randomly selected for each of the six replicates. ^a^AS, Agilent solution; NA, NimbleGen array; NS, NimbleGen solution; r1 and r2 are two replicate experiments for each platform. FR, flanking region; TR, targeted region.

### Genotype sensitivity

**Figure 2:**
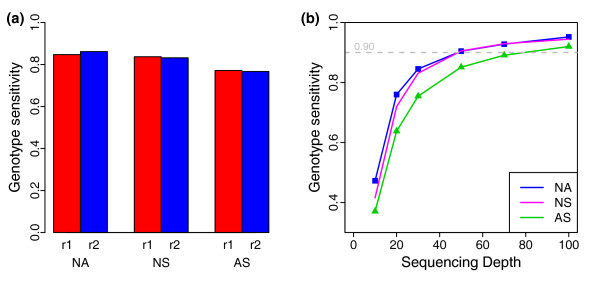
**Genotype sensitivity**. **(a) **Genotype sensitivity of six replicates at 30× sequencing depth. **(b) **Genotype sensitivity as a function of sequencing depth. For the analyses, subsets of reads from two combined replicate datasets for each platform were randomly extracted at different average depths. NA, NS and AS represent NimbleGen Sequence Capture Arrays, NimbleGen SeqCap EZ and Agilent SureSelect, respectively, while r1 and r2 are two replicate experiments for each platform.

Although the coverage of >99% of each targeted region of more than one-fold using all data sets an upper boundary for exome capture sensitivity for each replicate, only a proportion of these sites gained high-quality genotype assignments. To characterize this issue, we compared the genotype sensitivity in the 30× data sets (Figure 2a) using the criterion of >10-fold coverage and Phred-like quality >30. In these analyses, all three platforms showed very high genotype sensitivity (>77%); but, in comparison, the two NimbleGen platforms showed 6 to 8% higher (>83%) genotype sensitivity than the Agilent platform (approximately 77%), which is consistent with their better uniformity in coverage depth.

To obtain a more comprehensive insight, we further analyzed genotype sensitivity at other sequencing depths (Figure 2b) by randomly sampling from the combined sequencing data of the two replicates for each platform. Overall, the genotype sensitivity improved for all three platforms in a similar way as sequencing depth increased, and reached as high as >92% at approximately 100-fold coverage. The genotype sensitivity of the two NimbleGen platforms was often higher than the Agilent platform at a given sequencing depth. For example, genotype sensitivity was between 72% and 91% for the NimbleGen platforms at the usual sequencing depth of 20- to 50-fold, while it was 64 to 85% for the Agilent platform. Of interest, the curves of the two NimbleGen platforms nearly overlapped when sequence coverage depth was >30-fold. This indicates that these two platforms, which share a common set of DNA capture probes, have good inter-comparability.

We also analyzed genotype sensitivity at flanking regions; better NimbleGen results further emphasized the importance of the flanking regions for NimbleGen. From the above, we conclude that all three platforms had high genotype calling sensitivity at >30-fold coverage (>77%), with NimbleGen platforms showing slightly better performance.

### Reproducibility

**Figure 3:**
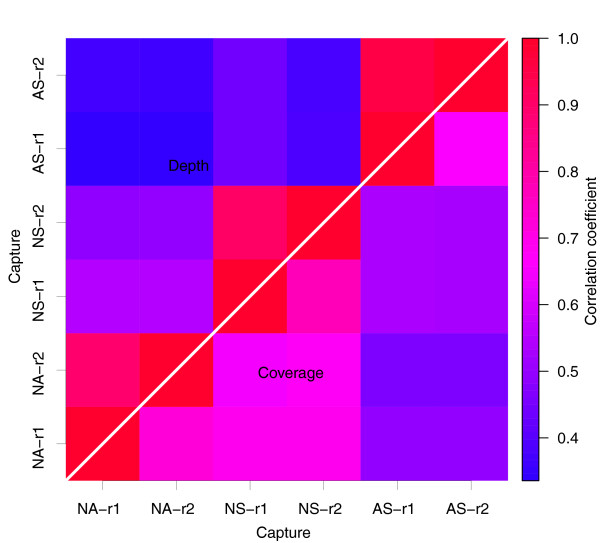
**Correlation of sequencing depth and coverage rate on consensus targeted CCDSs**. The graph shows pair-wise Pearson correlation coefficients for both sequencing depth (top-left triangle) and coverage rate (bottom-right triangle) based on the 182,259 CCDSs targeted by both Agilent and NimbleGen. NA, NS and AS represent NimbleGen Sequence Capture Arrays, NimbleGen SeqCap EZ and Agilent SureSelect, respectively, while r1 and r2 are two replicate experiments for each platform.

Technical reproducibility reflects the consistency of performance of each exome capture platform. Using the replicates for each of the three exome capture platforms, we determined the level of reproducibility within each platform. In considering inter-platform comparability as well, our evaluation focused on the set of targets that were shared between all three platforms (totaling 182,259 consensus coding sequences (CCDSs) covering 25,392,537 bp). This accounted for 70.1% and 66.1% of sensitivity in the NimbleGen and Agilent targeted regions, respectively. Using the approximately 30× data set, we analyzed the correlation of both coverage rate and mean depth on the CCDSs between any two of the six replicates (Figure 3). Each platform showed high intra-platform reproducibility (correlation coefficient at >0.65 for coverage rate and >0.90 for depth). The lower correlation coefficient for coverage rate (0.65 to 0.78) than for mean depth (0.90 to 0.96) was not surprising since the two correlations reflect different aspects of the data - that is, the quantitative sequencing depth and qualitative sequence coverage. For the inter-platform comparison, the two NimbleGen platforms showed higher correlation for both coverage rate and mean depth than the Agilent platform. This is consistent with the fact that the two platforms share a common set of DNA capture probes. These results together indicate generally high and comparable technical reproducibility of the three methods.

### GC bias and reference allele bias

Base composition has been shown to have a systematic effect on capture performance [13]. To explore this effect, we plotted mean sequencing depth against GC content. All three platforms showed biases against extremely low GC content (<20%) and high GC content (>75%), and the best coverage for GC content of 40 to 60% (Figure S4 in Additional file 2). However, we also observed a better coverage for the NimbleGen array platform, which had a better coverage of low GC content sequences without reduced coverage of the best-covered GC content. Thus, extreme GC content still poses a challenge for exome capture, but the chip-hybridization method (NimbleGen array platform) would likely be a better choice for targeted capture of genomic regions with lower GC content.

The allelic status of the probe sequences could also influence allelic capture efficiency at heterozygous sites, especially in situations where there are a large number of novel alleles being interrogated by exome capture. This occurs because the probes match the reference sequence and might capture perfectly matching library fragments better. To explore the impact of allelic status on the different platforms, we compared the ratio of reference allele depth to total depth for heterozygous sites in each exome capture with that in YanHuang whole-genome shotgun sequencing (WGSS). All three platforms showed consistent and significant biases towards the reference allele in capture (Figure S5 in Additional file 2), whereas WGSS did not have this bias. These results emphasize the need to account for the effect of reference allele bias in exome sequencing of tumors, in which acquired somatic mutations at any frequency may occur.

### Non-covered sequences

Even at 100-fold sequencing depth, a small proportion of the target region was still not covered by each platform. To gain insight into this issue, we analyzed the base composition of these missed sequences. In total, 97,654 to 190,318 sequences (0.29 to 0.56% of two targeted regions) were not covered at all by the combined full sets of data for each platform. Of these sequences, 19,803 (10 to 20% of the non-covered sequences) overlapped in all three platforms, and 71,257 (33% and 70% of the non-covered sequences) overlapped between the two NimbleGen platforms. The GC content was >72% for Agilent, >80% for NimbleGen Array, >79% for NimbleGen EZ, and 76% for all shared sequences. Thus, at very high sequencing depth (approximately 100×), the non-covered sequences for all three platforms were biased towards extremely high GC content.

### SNP detection

Given that exome capture is used primarily to identify genetic variants, we compared the SNP detection power among the three platforms. To do so, we called SNPs in the targeted regions together with 200-bp flanking sequence at high quality genotype-assigned sites in each of the approximately 30× data sets, and annotated them using the combined gene set used in the target annotation. Each platform detected roughly 25,000 to 40,000 SNPs, of which the largest group was from intronic regions, followed by synonymous SNPs and then non-synonymous SNPs, and finally by other categories (Table S4 in Additional file 1). The over-representation of intronic SNPs was more marked for the two NimbleGen platforms, where it provided over 10,000 more SNPs (35,000 to 40,000 in all) than the Agilent platform (25,000). Given the use of the same DNA and the similar proportion of intronic regions between the NimbleGen and Agilent platforms, this seems to be largely associated with the increased efficiency of capture by the NimbleGen platforms, especially in the flanking sequences. However, for synonymous and non-synonymous SNPs, which together represent the most functionally important groups, the Agilent and NimbleGen data showed substantial overlap and nearly similar levels of SNPs per gene to whole genome re-sequencing of the same individual. Thus, the three platforms could interrogate a similar high level of SNPs within protein-coding sequences in their targeted genes, which harbor changes that are most likely to have a functional impact.

### Accuracy of genotype and SNP calling

To assess their accuracy, we compared the genotypes and SNPs from each replicate (30× data) of the three platforms with those from Illumina 1 M beadchip genotyping and WGSS (about 36×) from the YanHuang project [26]. For better data comparability, we also derived genotypes for the WGSS using the same software and criteria as for the exome capture (see Materials and methods).

In comparison with the Illumina 1 M beadchip genotyping, which includes 1,040,000 successfully typed sites, each replicate showed approximately 39,000 to approximately 51,000 overlapping sites depending on the platform, and showed an overall genotype concordance of >99.81% for these sites (Table 3). In addition, each platform also achieved a similar high concordance rate with those variant sites found by chip genotyping, with >99.51% for all the SNP sites, and >99.56% for non-reference homozygous sites, and of particular note, even >99.48% for heterozygous sites, the genotypes of which are more difficult to assign than homozygous sites (Table 3). Relatively, the concordance of chip genotyping to the variant sites in each exome capture was also high, with >99.81% for all the SNP sites, and >99.88% for non-reference homozygous sites, and >99.71% for heterozygous sites (Table 3). These comparisons give a maximum estimate of both the false negative rate and false positive rate of <0.52% for the three exome captures.

**Table 3:** Concordance of genotypes and SNPs

	Concordance with 1 M bead genotyping data							Concordance with WGSS data						
**Replicate**^**a**^	All genotypes	SNPs in 1 M chip	SNPs in exome capture	Homs in 1 M chip	Homs in exome capture	Hets in 1 M chip	Hets in exome capture	All genotypes	SNPs in WGSS	SNPs in exome capture	Homs in WGSS	Homs in exome capture	Hets in WGSS	Hets in exome capture
NA-r1	99.846	99.641	99.826	99.649	99.987	99.633	99.687	99.999	99.216	98.636	99.951	99.868	98.683	97.750
NA-r2	99.854	99.670	99.835	99.708	99.975	99.637	99.714	99.999	99.264	98.616	99.943	99.850	98.768	97.724
NS-r1	99.854	99.679	99.819	99.682	99.951	99.676	99.707	99.998	99.211	98.396	99.974	99.747	98.657	97.426
NS-r2	99.849	99.660	99.841	99.684	99.987	99.640	99.716	99.999	99.197	98.706	99.979	99.752	98.629	97.949
AS-r1	99.816	99.526	99.823	99.571	99.948	99.486	99.712	99.998	98.783	98.021	99.917	99.824	97.945	96.703
AS-r2	99.815	99.514	99.805	99.556	99.880	99.477	99.738	99.998	98.762	97.972	99.927	99.771	97.893	96.645

For each replicate, the 30-fold data set used for Table 2 analyses was also used for the analyses. ^a^AS, Agilent solution; NA, NimbleGen array; NS, NimbleGen solution; r1 and r2 are two replicate experiments for each platform. Hets, heterozygotes; Homs, homozygotes.

In contrast, the two NimbleGen and Agilent datasets overlapped at 48,000,000 sites (with 83.8% sensitivity in targets) and 34,500,000 sites (with 76.2% sensitivity in targets) with WGSS genotypes, respectively. The substantially higher overlap of NimbleGen was attributed to its greater intronic content. This time, each exome capture platform showed a concordance of >99.999% for all overlapping sites, but >99.20% for all SNP sites, >99.92% for the homozygous non-reference sites and >97.90% for the heterozygous sites found in WGSS (Table 3). In comparison, the relative concordance of WGSS to the variant sites called in each exome capture was >97.97% for all SNP sites, >99.75% for the homozygous non-reference sites, and in particular was reduced to >96.65% for the heterozygous sites (Table 3), which is still acceptable. Note that for the heterozygous sites, compared to NimbleGen, Agilent showed approximately 1% reduction in concordance. In these analyses, cell-line DNA (approximately 40 generations) derived from lymphoblasts was sequenced using a read length of 90 bp, while for WGSS reads of 36 bp in length were generated from whole blood DNA. Thus, cell-line mutations, and errors due to increased sequencing length (errors accumulate with sequencing length) in the study may account for part of the decrease in concordance. Based on these results, the general false positive and false negative rate of each exome capture platform for SNP detection was <3.4% and <1.0%, respectively.

Taken together, these results indicate that although slight differences could be observed, accuracy was both high and comparable among the three platforms.

### Detection of medically interesting rare mutations

To further explore the power of the three exome capture platforms at identifying disease-causing rare mutations, we modeled the performance of each with the SNP set present in HGMD (Professional 2009.2) but absent from the 1000 Genomes Project database (BGI in-house data) (Table 4). Of the 39,906 mutations representing 1,931 diseases genes, both Agilent and NimbleGen targeted >95.8% sites, and showed >93.4% sites with at least 1× coverage and genotype sensitivity of >79% sites (>10× coverage and >Q30) at 30× sequencing depth. But in comparison, Agilent targeted more sites (98.5% compared to 95.8%), and correspondingly showed approximately 1.5% more covered sites (>1× coverage; 95.1% compared to 93.4%) than NimbleGen. In contrast, NimbleGen (the best performance was with NimbleGen Array Capture) showed 1.4% more genotype sensitivity (80.4% compared to 79%), and 3.6% less low quality coverage sites or uncovered sites (15.2% compared to 18.8%) than Agilent. The number of known potentially disease-causing SNPs detected ranged from 14 to 19 (Table 3). These observations are consistent with the larger targeted gene set of Agilent, and the higher capture efficiency of NimbleGen. Thus, the analyses demonstrated the very high power of the three exome capture platforms for identifying medically interesting rare mutations.

**Table 4:** Power for identifying disease-causing rare mutations

	NA-r1	NA-r2	NS-r1	NS-r2	AS-r1	AS-r2
High quality genotype assigned sites	32,139	32,674	31,750	31,923	31,685	31,353
Reference genotypes	32,124	32,658	31,732	31,909	31,666	31,335
SNPs	15	16	18	14	19	18
Low quality genotype assigned sites	6,064	5,529	6,453	6,280	7,349	7,681
Uncovered	1,703	1,703	1,703	1,703	872	872

For each replicate, the 30-fold data set used for Tables 2 and 3 analyses was also used for this analysis. AS, Agilent solution; NA, NimbleGen array; NS, NimbleGen solution; r1 and r2 are two replicate experiments for each platform.

### Performance on common targeted regions

Hitherto, most of the comparisons have been based directly on the current versions of the three platforms, which may not reflect only the intrinsic differences in performance among the three methods, but also the differences in content. To address this issue, we compared key performance parameters on the approximately 30 Mb of targeted regions in common (83.3 Mb with flanking sequences; Table S1 in Additional file 1). For specificity, we found that each replicate of the three platforms showed a somewhat reduced unique mapping rate of >44% filtered reads to the common targeted regions, and that the two NimbleGen platforms achieved, on average, a 12% higher unique mapping rate than the Agilent platform when including the 200-bp flanking sequences in the analyses (Table S5 Additional file 1). This result is consistent with the initial analyses above.

For uniformity and sensitivity, we also found that each platform showed very similar performance to that above, and that the two NimbleGen platforms performed better than the Agilent one (Table S5 in Additional file 1). For example, at a sequencing depth of 30×, NimbleGen had, on average, approximately 6% higher genotype sensitivity than Agilent (85% compared to 79%). For SNP detection, the detection level of each SNP category in each platform, including the greater detection of intronic SNPs (and thus the total SNP number) by the NimbleGen platforms (>13,000 more SNPs than Agilent, >35,000 compared to approximately 22,000), was also similar to the analyses above (Table S4 in Additional file 1); but in comparison, despite general inter-comparability, the two NimbleGen platforms detected approximately 400 more coding SNPs (12,400 compared to 12,000) in the common targeted regions while the Agilent platform detected approximately 900 more coding SNPs elsewhere (13,500 compared to 12,600) (Table S4 in Additional file 1). This difference could be explained by the fact that NimbleGen had a better capture efficiency while Agilent targeted an approximately 4-Mb larger region and correspondingly 1,000 more genes.

Finally, for the accuracy of SNP detection and genotypes, we also observed similar false positive and false negative rates for each platform at 30× coverage (Table S6 in Additional file 1) to that in whole dataset in comparison with the data from array genotyping and WGSS. Thus, we conclude that each platform was highly consistent in performance in the common targeted region analyses here compared with the analyses of the entire content above, which is not surprising given the high overlap (Agilent, 30 Mb/34.1 Mb ≈ 80%; NimbleGen, 30 Mb/40 Mb ≈ 88%).

## Discussion

In this study, we present a comprehensive comparison of three widely adopted human whole-exome capture platforms from two manufacturers. Since the three platforms, in principle, represent the three classes of exome capture technologies currently available, data on their performances likely also reflect the intrinsic power and limitations of exome capture as a technology.

For the current versions of the three platforms, the number of targeted genes and their CD coverage rate are important considerations for human genetic studies. Although most well-annotated human genes (>76%) were targeted by all three platforms, Agilent sought to target a larger set of genes (approximately 1,000 more protein-coding genes and approximately 100 more microRNA genes) and thus provided a better coverage of protein-coding sequences. In contrast, NimbleGen emphasized a more important role for flanking regions in capture probe design, and, in practice, had a greater number of genes with a high rate of CD coverage (Figure S6 in Additional file 2) due to better capture efficiency.

Exome capture efficiency is another important factor for comparison of capture platforms. In our hands, we observed that the two NimbleGen platforms showed better capture efficiency than the Agilent platform. Specifically, the two NimbleGen platforms showed approximately 10% higher capture specificity with the expanded targeted regions (66.6% compared to 58.3%), better uniformity of coverage, and 3 to 7% more sensitivity in genotype assignment (83 to 95% compared to 76 to 92% over the range 30× to 100× coverage of targeted regions). Thus, a lower sequencing depth was required for NimbleGen platforms for a given genotype sensitivity on targeted regions, which can impact experimental cost.

The ability to identify SNPs in protein-coding sequences, especially those medically interesting rare mutations, which ultimately measures the power of exome sequencing, was another important consideration. Despite general inter-comparability (12,500 to 13,500 SNPs), we found that, at the same sequencing depth (30×), NimbleGen detected a more complete set of SNPs (approximately 400 more SNPs) than Agilent for the common targeted coding sequences due to better exome capture efficiency, but the Agilent platform could detect more SNPs (approximately 900 SNPs) in total number due to its larger number of targeted genes. Similarly, for identifying medically interesting rare mutations, we found in model analyses that all three platforms not only showed similar high power at 30× sequencing depth in interrogating known HGMD mutations filtered to remove 1000 Genomes Project variants present in the general population, but the small differences reflected the general features of each platform (Agilent could target 1.8% more, and cover 1.5% more mutation sites, but NimbleGen showed 1.4% more mutations with high quality genotype assignment).

Input DNA amount, the convenience of conducting experiments and the cost of reagents will also be important considerations. Especially, the amount of DNA required for each method itself will impact cost as well as the ease of carrying out experiments, and is a major consideration for precious biological samples with limited availability. In these senses, the two solution hybrid platforms, Agilent and NimbleGen EZ, showed great advantages over the chip hybridization platform. These two solution-based platforms require smaller amounts of input DNA (approximately 3 μg) and no specialized equipment. In addition, reagent costs for these two platforms are lower when more than ten samples are being studied, and could possibly be further reduced with the introduction of sample pooling prior to the capture possess.

For performance aspects, such as the accuracy of SNP detection, GC bias and reference allele bias, and reproducibility, we did not observe great differences among the three platforms.

Taken together, our results here demonstrate that although the three platforms showed general comparability of performance, the two solution hybrid platforms would be the leading choice for most studies, especially those using large numbers of samples. In comparing these two, the Agilent platform showed a larger set of targets, targeting a more comprehensive set of human protein-coding genes and providing more complete coverage of their CDs, while the NimbleGen platform had better capture efficiency and could provide a higher proportion of CDs with high quality genotype assignments (thus higher completeness of SNP detection), and required lower sequence coverage because of its greater evenness. Thus, a choice between the two platforms is surprisingly difficult: both are highly effective and the number of targeted genes, their CD coverage, genotype sensitivity and sequencing amount/cost required must be balanced. The larger number of genes targeted by Agilent provides an overall advantage in the versions used here, but it is important to point out that both NimbleGen and Agilent are making great progress in target design. For example, in the latest (July 2011) versions, both target sets have been expanded (NimbleGen EZv.20 to 44 Mb, Agilent to 50 Mb), and currently cover more than 90% of annotated human genes (Table S7 in Additional file 1).

## Conclusions

We demonstrate here a systematic evaluation of the performance of the current versions of three human whole-exome capture platforms. The data reported here will make it easier for researchers to more carefully assess the type of exome capture technology that will work best for their experimental goals and costs, and allow them to improve their own experimental design to take advantage or reduce the limitations of the available platform types.

## Materials and methods

### Genomic DNA and kit preparation

Genomic DNA was extracted from a lymphoblastoid cell line of YanHuang [26] using proteinase K and phenol/chloroform [27] and further subjected to RNase treatment. DNA sample quality and quantity were initially characterized by gel electrophoresis and nano-drop measurement, and further quantified using the Quant-iT dsDNA HS Assay kit (0.2 to 100 ng; Invitrogen, Q32854, Carlsbad, CA, USA). NimbleGen Sequence Capture Array (Human Exome 2.1 M Array) and SeqCap EZ (v1.0) kits, and Agilent SureSelect kits (Human All Exon Kits) were purchased from their respective manufacturer.

### Exome capture library preparation

Prior to library construction, we optimized the manufacturers' standard protocols for our sequencing pipeline. Major parameters for optimization included input DNA quantity, fragmentation size, number of PCR cycles and indexing system. As a result, we adopted the following protocol.

Input DNA quantities of 10 μg, 3 μg and 3 μg were used for NimbleGen Sequence Capture Array, NimbleGen SeqCap EZ and Agilent SureSelect library preparation, respectively. The DNAs were fragmented to 200 to 250 bp in size, followed by end-repair, A-tailing and BGI paired-end index adapter ligation, following the Illumina DNA library preparation protocol described elsewhere [28]. Four cycles of pre-capture amplification were then conducted with Platinum Pfx DNA polymerase (Invitrogen) under the PCR conditions: 2 minutes at 94°C; four cycles of 10 s at 94°C, 30 s at 62°C, and 30 s at 72°C; then 300 s at 72°C. PCR products were further analyzed using a Bioanalyzer (Agilent, Santa Clara, CA, USA), and quantified by Qubit BR measurement (Invitrogen) before performing exome capture.

Exome capture was performed with the PCR products following each of the three manufacturers' standard protocols. Then, ten cycles of post-capture amplification were conducted using Platinum Pfx DNA polymerase (Invitrogen) under slightly modified PCR conditions compared to pre-capture amplification (2 minutes at 94°C; 10 cycles of 10 s at 94°C, 30 s at 58°C, and 30 s at 72°C; then 300 s at 72°C). Final libraries were validated by Bioanalyzer analysis (Agilent) and quantitative PCR, in preparation for massively parallel sequencing.

### Sequencing and pre-mapping data process

Sequencing of each library was done on an Illumina HiSeq2000 as paired-end 90-bp reads (PE90) after indexing individual libraries and pooling them in pairs. Each library was initially sequenced to a depth providing an approximately 30-fold mapped coverage on targeted regions, and then one of the two replicates was further chosen from each of the three platforms to sequence to >60-fold coverage on targeted regions. Raw image files were processed by the Illumina pipeline (version 1.3.4) for base calling and to generate a raw read set. Adapter contamination and reads of low quality (more than four 'N' nucleotides) were identified and removed before mapping.

### Mapping, genotype and SNP calling

SOAP (v2.21) [29] was used to align the reads to the NCBI human genome reference assembly (build 36.3) with parameters set to '-a -b -D -o -u -t -l 35 -n 4 -r 1 -2 -v 2 -s 40'. These settings provided the best mapping rate by giving highest priority to paired-end and then lower priority to unpaired single-end matches. SOAPsnp [30] (v1.03) was used to call consensus genotypes with the parameters '-i -d -o -r 0.00005 -e 0.0001 -t -s -2 -u -M -L -T', where '-T' used the targeted and flanking regions. As our sample is from a male, we added the parameter '-m' for the sex chromosomes. Then SNPs were extracted from the consensus genotype file, and those with sequencing depth <10, quality <30, copy number of a nearby region >2, or other SNPs within 5 bp were filtered out to get high-confidence calls.

### Data accessibility

All data described here are being deposited to the NCBI Sequence Read Archive [SRA:035389].

## Abbreviations

bp: base pair; CCDS: consensus coding sequences; CD: coding sequence; Gb: billion base pairs; HGMD: Human Gene Mutation Database; Mb: million base pairs; PE90: paired-end 90-bp read; SNP: single nucleotide polymorphism; WGSS: whole-genome shotgun sequencing.

## Competing interests

The authors declare that they have no competing interests.

## Authors' contributions

XZ and HY designed the study. YuX, JiaW and TJ analyzed all data. JiW, JuW, MW, XL, GT and HJ developed the optimized exome capture protocols, and conducted library preparation and sequencing. A designed the study, coordinated all work and wrote the manuscript. CTS designed the study and revised the manuscript. YaX revised the manuscript. All authors read and approved the final manuscript.

## Supplementary Material

Additional file 1 **Supplementary Tables 1 to 7**. Click here for file

Additional file 2 **Supplementary Figures 1 to 5**. Click here for file
