# Supplementary material for: Comprehensive comparison of three commercial human whole-exome capture platforms
Source: Genome Biol. 2011 Sep 28;12(9):R95. doi: 10.1186/gb-2011-12-9-r95 (PMC3308058; doi:10.1186/gb-2011-12-9-r95)
Supplement: Additional file 1 — Supplementary Tables 1 to 7. [file gb-2011-12-9-r95-S1.PDF]

Supplementary Table 1 - A comparison of two Agilent and Nimblegen targets

	Targets size (bp)	Targets and flanking sequences size (bp)	# genes	# CCDs
Agilent	37,640,396	94,877,198	17,199	199,798
NimbleGen	34,108,810	87,281,680	16,188	184,462
Common	30,046,886	83,257,316	15,883	182,238

Supplementary Table 2 – Annotation of current versions of Agilent (38M) and NimbleGen (34M) targets

		Agilent 38M				NimbleGen 34M				Total
		**Annotated elements**	**Percent of all**	**Target length (bp)**	**Percent of all**	**Annotated elements**	**Percent of all**	**Target length (bp)**	**Percent of all**	**Annotated elements**
**Protein coding genes**		17,199	80.6	NA	NA	16,188	75.9	NA	NA	21,326
	CDS	199,798	81.9	27,499,258	73.06	184,462	75.6	24,272,070	71.2	244,069
	5-UTR	20,339	79.0	575,540	1.5	18,979	73.7	577,794	1.7	25,746
	3-UTR	20,318	78.9	566,558	1.5	19,048	74.0	510,893	1.5	25,746
	intron	179,975	82.4	8,925,625	23.7	166,237	76.1	8,680,914	25.5	218,323
	transcripts	20,614	80.1	NA	NA	19,466	75.6	NA	NA	25,746
**MicroRNA genes**		658	91.4	30,615	0.1	550	76.6	27,540	0.1	718
**Other**		NA	NA	42,800	0.1	NA	NA	39,599	0.1	NA

Protein-coding genes were annotated using merged data from 21,326 genes from the CCDS, refGen and EnsemblGen databases, while micro RNA genes included 719 genes from the human microRNA database. 200-bp flanking both ends of the targeted sequences were included to obtain the most comprehensive annotation set. The 5'-UTR and 3'-UTR were defined as the upstream and downstream regions flanking of the first/last CDS region, respectively.

Supplementary Table 3- Statistics for NimbleGen and Agilent targeted genes

	# genes in database	Nim & Agi common targeted genes	Nim_specific	Agi_specific
Diseases genes	5231	4612(88.2%)	24(0.5%)	261(5.0%)
HGMD	2273	2112(92.9%)	2(0.1%)	79(3.5%)
CGP	408	366(89.7%)	1(0.2%)	17(4.2%)
GWAS	2491	2139(85.9%)	17(0.7%)	136(5.5%)
OMIM	2883	2596(90.0%)	7(0.2%)	129(4.5%)
Total genes	21326	15883(74.5%)	305(1.4%)	1316(6.2%)

Supplementary Table 4 – The power of SNPs detection by the three platforms in individual targeted regions and common targeted regions

	NA-r1	NA-r2	NS-r1	NS-r2	AS-r1	AS-r2
**All targets and their flanking regions**						
Coding_synonymous	6524	6770	6757	6715	7135	7002
Coding_nonsynonymous	5864	6015	6055	5766	6460	6380
Missense	5539	5684	5714	5442	6142	6063
Nonsense	37	39	46	40	42	37
Readthrough	7	5	4	5	7	7
Splice site	281	287	291	279	269	273
Intron	22106	20704	24349	22349	10043	9903
5' UTRs	881	854	1081	1010	547	531
3' UTRs	1078	1011	1285	1141	612	611
Intergenic	91	73	132	112	83	79
Total	36544	35427	39659	37093	24880	24506
**Common targets and their flanking regions**						
Coding_synonymous	6425	6666	6652	6620	6453	6332
Coding_nonsynonymous	5767	5918	5950	5675	5678	5603
Missense	5447	5591	5614	5355	5396	5322
Nonsense	33	36	42	37	35	30
Readthrough	7	5	4	5	6	6
Splice site	280	286	290	278	241	245
Intron	21785	20423	23926	21939	9022	8862
5' UTRs	875	849	1067	1003	496	478
3' UTRs	1056	992	1257	1113	554	549
Intergenic	79	64	102	87	15	14
Total	35987	34912	38954	36437	22218	21838

For the analyses, data that have ~30 fold coverage on targeted regions or on common targeted regions were used.

Supplementary Table 5 - Specificity, uniformity and sensitivity of the three platforms on common targeted regions

	AS		NA		NS	
	r1	r2	r1	r2	r1	r2
**Specificity**						
% reads mapped uniquely to TR	48.73	45.68	46.65	44.16	44.89	46.09
% reads mapped uniquely to TF	54.38	50.93	65.79	60.81	65.89	65.67
**Uniformity (30x data)**						
On TR						
% bases uncovered	1.84	1.95	1.05	0.72	1.37	1.17
% bases with <10x coverage	19.36	19.73	13.71	12.56	14.71	15.32
% bases with 10-50x coverage	59.03	58.51	68.92	72.9	68.52	67.31
% bases with >50x coverage	19.77	19.81	16.32	13.82	15.4	16.2
On FR						
% bases uncovered	41.91	42.4	5.99	6.39	5.33	5.84
% bases with <10x coverage	44.4	44.07	55.77	58.82	53.01	56.39
% bases with 10-50x coverage	12.44	12.31	37.46	34.28	39.8	36.15
% bases with >50x coverage	1.25	1.22	0.78	0.51	1.86	1.62
**Sensitivity**						
% high quality genotyped sites with 30x data	76.88	76.55	85.16	86.65	84.28	83.94
Sequencing-depth VS sensitivity						
% high quality genotyped sites with 10x data	38.84		48.07		42.61	
% high quality genotyped sites with 20x data	66.07		76.46		72.93	
% high quality genotyped sites with 30x data	77.59		84.97		83.85	
% high quality genotyped sites with 50x data	86.83		90.91		91.08	
% high quality genotyped sites with 70x data	90.49		93.25		93.42	
% high quality genotyped sites with100x data	93.09		95.64		94.91	

Supplementary Table 6 - concordance of genotypes and SNPs in common targeted regions (~30x data)

Replicate	Concordance with 1M bead genotyping data (%)							Concordance with WGSS (%)						
	All genotypes	SNPs in 1M chip	SNPs in exome capture	Homs in 1M chip	Homs in exome capture	Hets in 1M chip	Hets in exome capture	All genotypes	SNPs in WGSS	SNPs in exome capture	Homs in WGSS	Homs in exome capture	Hets in WGSS	Hets in exome capture
NA-r1	99.835	99.622	99.678	99.494	100.000	99.563	99.827	99.998	98.687	97.325	99.951	99.866	99.215	98.379
NA-r2	99.835	99.573	99.684	99.594	100.000	99.582	99.831	99.998	98.774	97.274	99.949	99.847	99.267	98.345
NS-r1	99.837	99.619	99.695	99.517	99.978	99.572	99.826	99.998	98.655	96.903	99.980	99.742	99.210	98.081
NS-r2	99.814	99.480	99.672	99.555	99.978	99.515	99.814	99.998	98.633	97.481	99.986	99.748	99.198	98.424
AS-r1	99.816	99.496	99.663	99.493	99.952	99.494	99.797	99.998	98.043	96.160	99.919	99.816	98.838	97.693
AS-r2	99.809	99.446	99.701	99.470	99.903	99.457	99.796	99.998	97.948	96.239	99.942	99.780	98.799	97.737

Supplementary Table 7 – Annotation of latest version of Agilent (50M) and NimbleGen (EZv2.0) targets

	Agilent 50M				NimbleGen EZv2.0				Total
	**Annotated elements**	**Percent of all**	**Target length (bp)**	**Percent of all**	**Annotated elements**	**Percent of all**	**Target length (bp)**	**Percent of all**	**Annotated elements**
**Protein-coding genes**	21,097	98.9	NA	NA	19,335	90.7	NA	NA	21,326
CDS	240,163	98.4	32,973,464	65.6	231,014	94.7	29,521,397	66.8	244,069
5-UTR	24,392	94.7	881,957	1.8	20,211	78.5	784,531	1.8	25,746
3-UTR	24,153	93.8	845,742	1.7	19,181	74.5	637,773	1.4	25,746
intron	216,213	99.0	13,902,934	27.7	207,785	95.2	11,947,354	27.0	218,323
Transcripts	25,448	98.8	NA	NA	23,361	90.7	NA	NA	25,746
**MicroRNA**	676	94.2	31,412	0.1	692	96.4	33,739	0.1	718
**Other**	NA	NA	1,602,838	3.2	NA	NA	1,289,920	2.9	NA

Both target sets were annotated using the same strategy as described in *Supplementary Table2*.
