# Supplementary material for: Comprehensive comparison of three commercial human whole-exome capture platforms
Source: Genome Biol. 2011 Sep 28;12(9):R95. doi: 10.1186/gb-2011-12-9-r95 (PMC3308058; doi:10.1186/gb-2011-12-9-r95)
Supplement: Additional file 2 — Supplementary Figures 1 to 5. [file gb-2011-12-9-r95-S2.PDF]

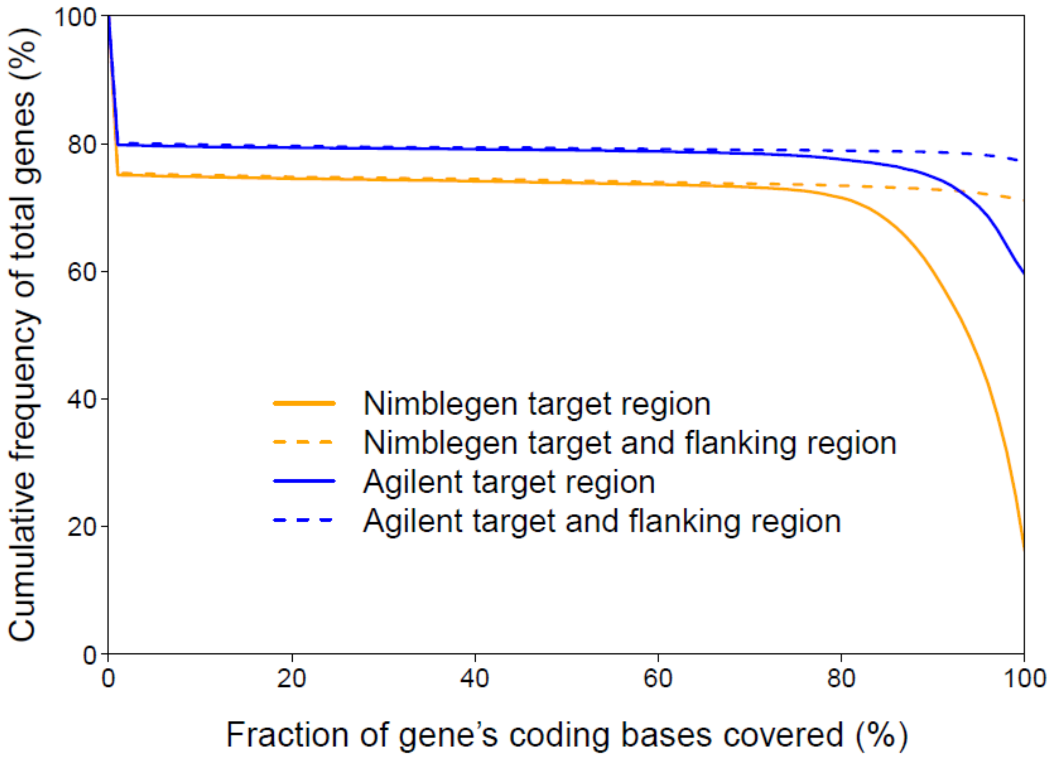

Figure S1 Completeness of protein-coding gene coverage by exome capture targets. The fraction of coding bases covered by targeted regions (solid line) or expanded targeted regions (dashed line) were calculated on a gene-by-gene basis, for both Agilent and NimbleGen. The cumulative, ordered fractions of genes that meet minimum criteria were plotted. The faction of genes was calculated based on merged data from 21,326 genes from the CCDS, Refgene and Ensembl gene data bases.

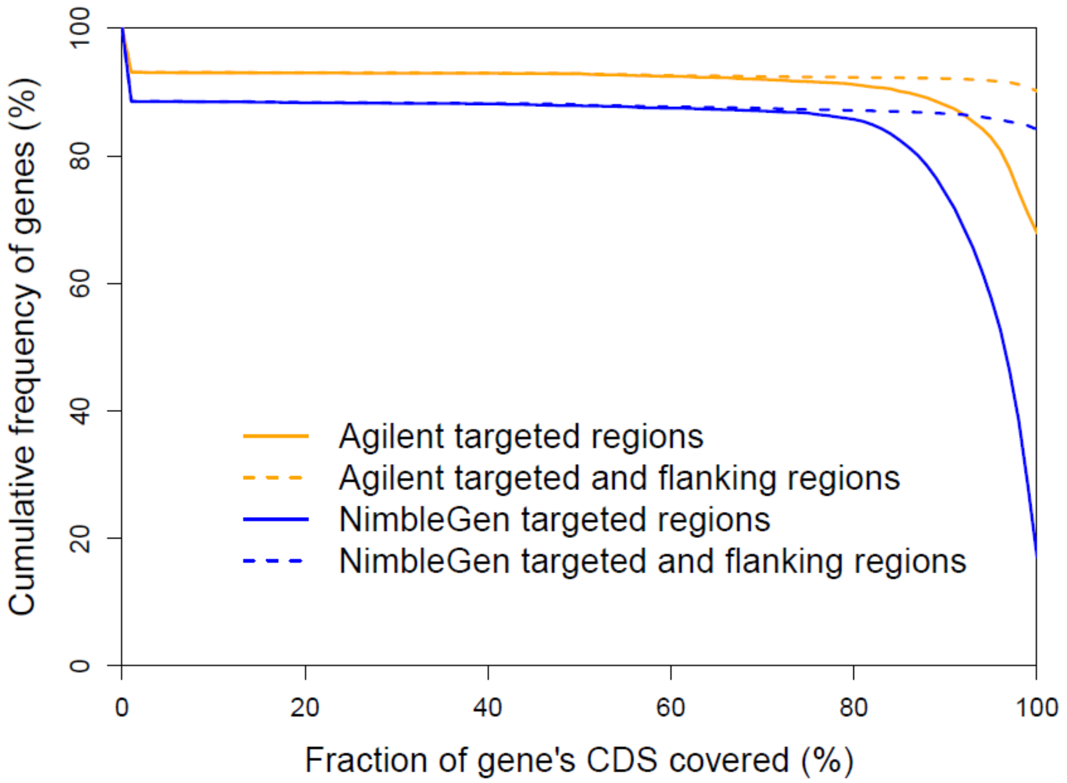

Figure S2 Completeness of protein-coding sequences coverage of known diseases genes. The fraction of coding bases covered by targeted regions (solid line) or expanded targeted regions (dashed line) were calculated on gene-by-gene basis, for both Agilent and NimbleGen. The cumulative, rank-ordered fractions of genes that meet minimum criteria were plotted. The faction of genes was calculated based on a combination of 5,231 unique genes from the OMIM, HGMD, GWAS and CGP databases.

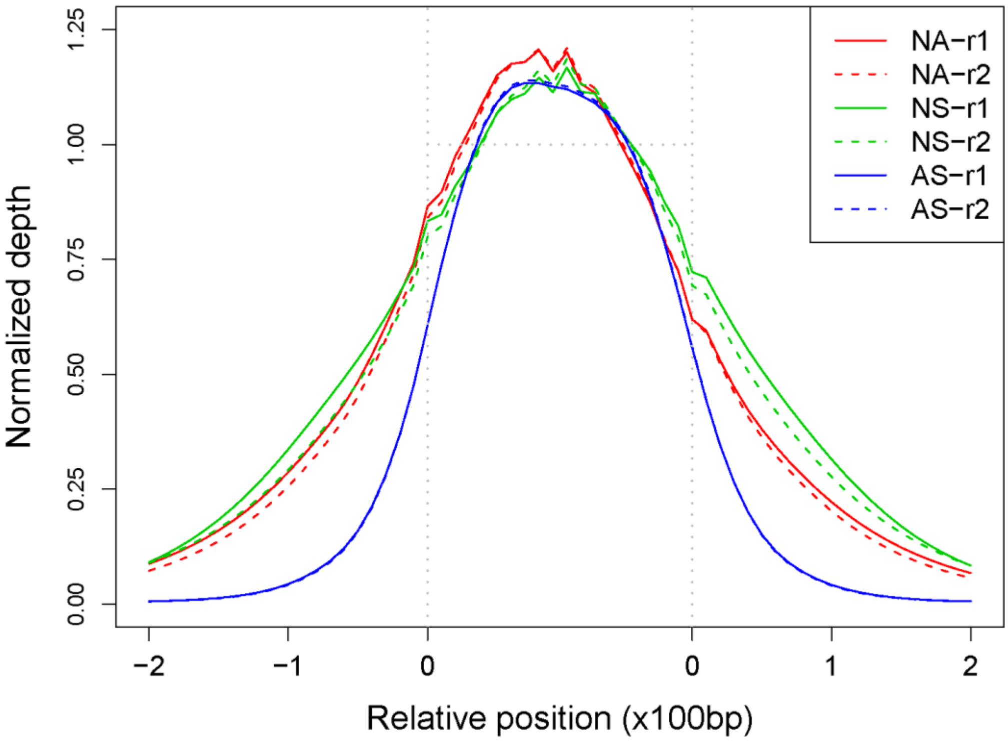

Figure S3 Coverage profiles of targeted regions. Each targeted region was split into 20 equally sized bins (each of about 10 bp in size), and mean sequencing depth was calculated for each bin. This process was also performed for 10-bp windows both 200 bp upstream (left) and downstream (right) of the targeted regions. If the distance of two neighboring targeted regions was <400 bp, the region was split into two equal sizes and half associated with each targeted region. For better comparison, sequencing depth for each platform was normalized by dividing by the average sequencing depth on targeted regions.

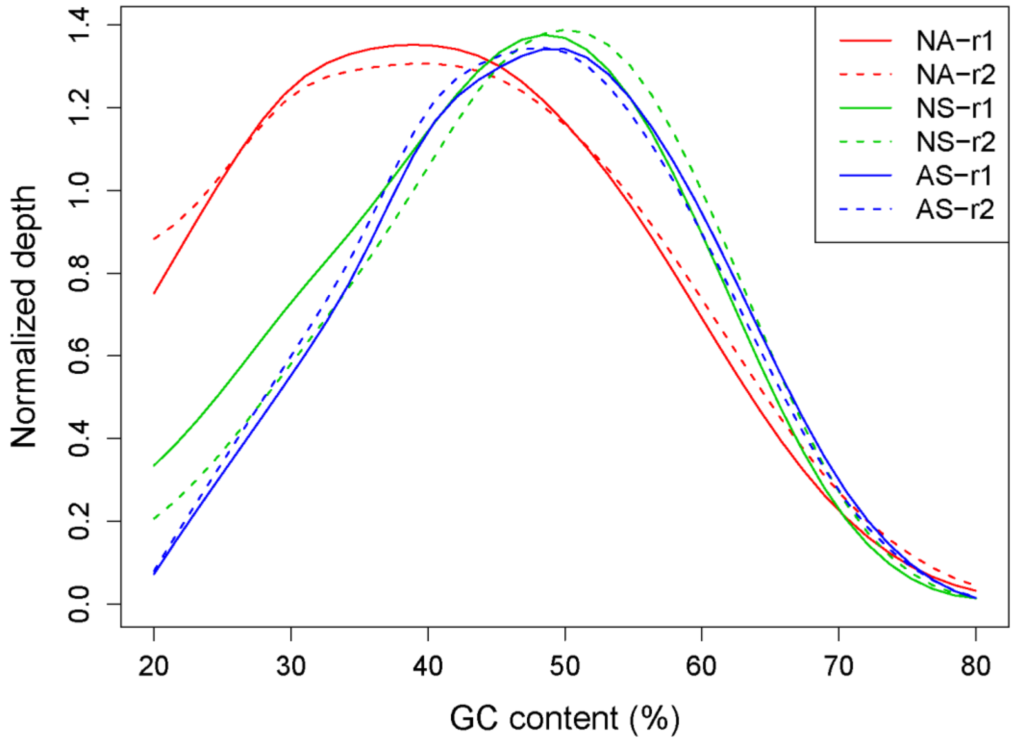

Figure S4 Normalized mean sequencing depth as a function of G+C content. Note that GC content and sequencing depth was calculated using each targeted region as a window.

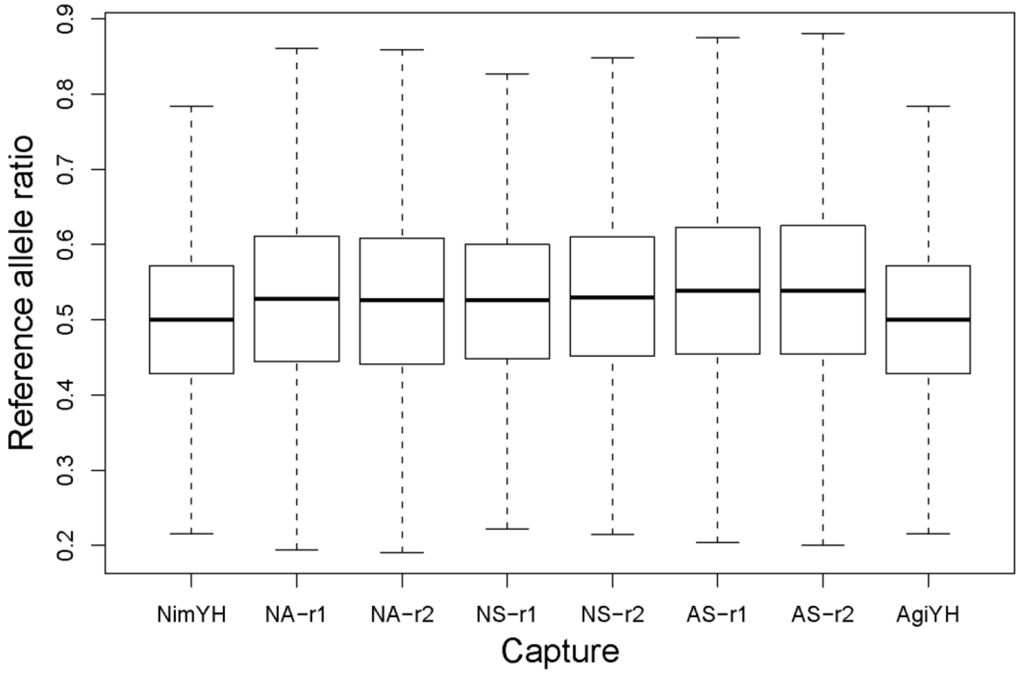

Figure S5 Reference-allele biases at heterozygous SNP sites. Shown is box-plot of the percentage of reference allele depth. The percentage of reference allele depth at each heterozygous site was calculated for each replicate of the three platforms as well as for whole genome sequencing within the targeted and flanking regions of Agilent (AgiYH) and NimbleGen (NimYH).

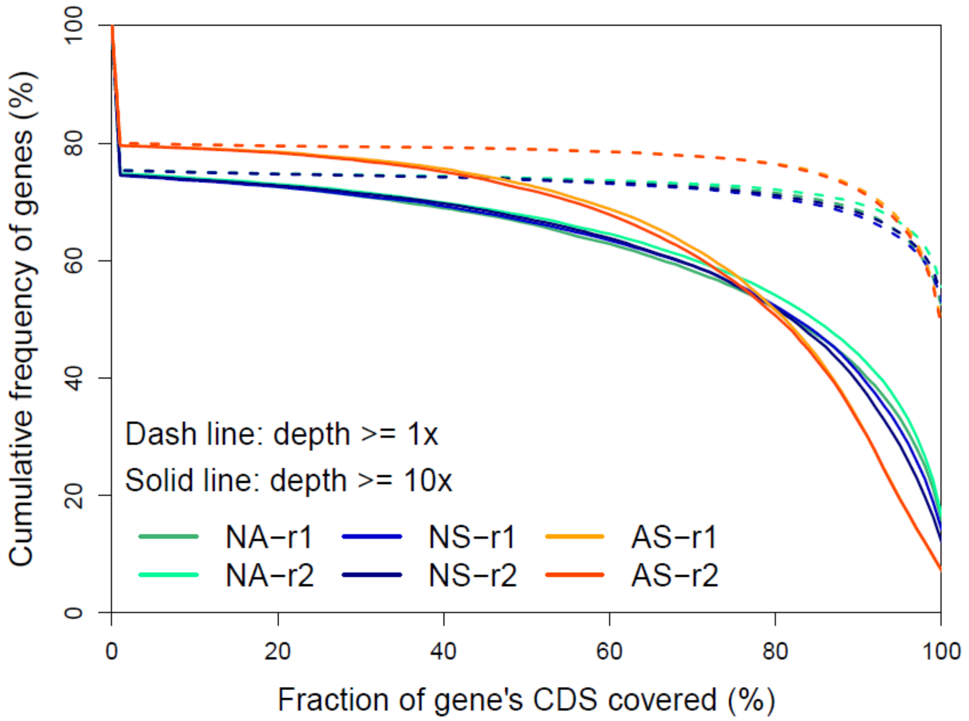

Figure S6 Completeness of protein-coding gene coverage by each replicate. We calculated for each replicate the fraction of coding bases covered at least 1x (dotted line) or 10x (solid line) on a gene-by-gene basis, for 21,326 genes merged from the CCDS, Refgene and Ensembl gene databases. The cumulative, ordered fractions of genes that meet minimum criteria are plotted above.
